# Modal Identification in an Automotive Multi-Component System Using HS 3D-DIC

**DOI:** 10.3390/ma11020241

**Published:** 2018-02-05

**Authors:** Ángel Jesús Molina-Viedma, Elías López-Alba, Luis Felipe-Sesé, Francisco A. Díaz

**Affiliations:** 1 Department of Mechanical and Mining Engineering, Campus Las Lagunillas, University of Jaén, 23071 Jaén, Spain; elalba@ujaen.es (E.L.-A.); fdiaz@ujaen.es (F.A.D.); 2 Department of Mechanical and Mining Engineering, Campus Científico Tecnológico de Linares, University of Jaén, 23700 Linares, Spain; lfelipe@ujaen.es

**Keywords:** HS 3D-DIC, automotive lighting system, multi-component, multi-material, experimental modal analysis, transmissibility functions

## Abstract

The modal characterization of automotive lighting systems becomes difficult using sensors due to the light weight of the elements which compose the component as well as the intricate access to allocate them. In experimental modal analysis, high speed 3D digital image correlation (HS 3D-DIC) is attracting the attention since it provides full-field contactless measurements of 3D displacements as main advantage over other techniques. Different methodologies have been published that perform modal identification, i.e., natural frequencies, damping ratios, and mode shapes using the full-field information. In this work, experimental modal analysis has been performed in a multi-component automotive lighting system using HS 3D-DIC. Base motion excitation was applied to simulate operating conditions. A recently validated methodology has been employed for modal identification using transmissibility functions, i.e., the transfer functions from base motion tests. Results make it possible to identify local and global behavior of the different elements of injected polymeric and metallic materials.

## 1. Introduction

In the automotive industry, there are significant phenomena and dynamics events—due to the engine, road friction, airflow turbulence, etc.—which must be taken into account to guarantee the structural integrity of different systems used in the vehicle. Special attention must be paid to resonances in the typical vibration spectrum that a vehicle undergoes. Considering the lighting system, the vibrational behavior of the different components have a major influence for the correct cut-off performance of the illumination parts. Thus, the design stages evaluate the influence of the material properties in the dynamic behavior of the ensemble supported by powerful numerical simulation conventionally based on the finite element method. Later on, the experimental validation of the predicted features must be performed. In this field, experimental modal analysis is an extended methodology that relies on the identification of the modal parameters, i.e., natural frequencies, mode shapes, and damping ratios [1]. However, the instrumentation of these systems is especially complex and typically is too invasive so that the results are not representative of the actual behavior.

In the latest years, different techniques such as scanning laser doppler vibrometry (SLDV) or high speed 3D digital image correlation (HS 3D-DIC) appeared as an alternative to invasive transducers. Laser vibrometry is an interferometric technique that employs a pointwise laser vibrometer to analyze a specific point on the specimen surface in the direction of the laser beam [2]. With the scanning system, this task is repeated at different points to build up a high spatial resolution characterization. A continuous scan allows operational deflection shapes characterization along a defined path [1,3,4]. Three lasers must be employed to perform 3D measurements. Although SLDV is extensively developed for experimental modal analysis, HS 3D-DIC provides higher spatial resolution and 3D measurements with a considerably cost reduction in the equipment [5,6,7]. Both features are especially interesting considering the 3D distribution and the shape complexity of the components of a lighting system. Different studies have tested the capabilities of HS DIC in the characterization of mode shapes by forcing a single resonance [5,8,9,10,11,12,13,14,15,16]. Full modal characterizations, i.e., for the three modal parameters, were already performed [6,7,17,18,19,20,21], using frequency response functions (FRF). These are transfer functions between a force excitation and a displacement response measured by DIC. However, in a lighting system, excitation is mainly transmitted from the whole vehicle structure through the attaching points. This is typically characterized as a base motion excitation problem. Experimentally, the excitation is registered as a motion instead of force in such cases. Hence, transfer functions are now named as transmissibility functions. Nevertheless, modal identification algorithms are mostly developed to manage FRFs, i.e., with force excitation. Ha et al. [22] performed a modal analysis using HS 3D-DIC in an artificial beetle wing using base motion excitation but employing transmissibility functions as if they were FRFs, assuming similitudes between them. Molina-Viedma et al. [23] proposed a methodology to properly perform modal identification in transmissibility functions obtained by HS 3D-DIC. In that work, a theoretical conversion from transmissibility functions to their equivalent FRFs is proposed under single-degree-of-freedom (SDOF) assumptions. The circle-fit approach [1,24] was employed for modal characterization as an efficient method to deal with the high amount of FRF data generated by DIC. The methodology was validated in a cantilever beam using theoretical and numerical models.

In the present work, this methodology is extended for modal characterization of a multi-component lighting system. This is the first time that this kind of methodology is employed for multi-component multi-materials specimens. The modal properties of the different materials can be extracted individually from a single test and evaluated according to the material type. Outstanding results are obtained regarding 3D mode shapes where the individual and ensemble motions were revealed. Therefore, it is shown a high potential to explore the mechanical behavior of the elements during working condition.

## 2. Materials and Method

**Figure 1:**
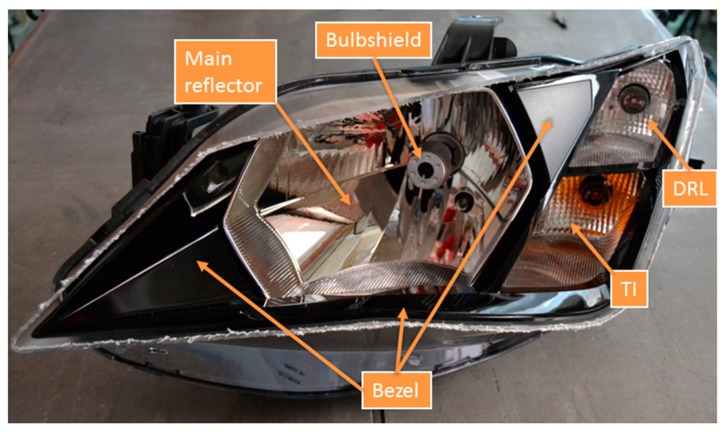
Left-hand headlamp specimen employed for experimental modal analysis using HS 3D-DIC.

The proposed multi-component modal analysis was developed in a left-hand halogen headlamp from a vehicle, shown in Figure 1. The headlamp shows different functional elements such us the bezel, the low and high beam reflector, hereafter mentioned as the ‘main reflector’, that contains a screwed bulbshield to fix the lamp, the turn indicator (TI), and the day running light (DRL) functions. The materials which these elements are made of are shown in Table 1. All components are clipped between them. This subassembly is finally fixed to the housing (polypropylene 40% talc) providing the boundary condition for the elements. To avoid reflection and refraction, the front part from the external lens (polycarbonate) in the headlamp was removed. 

### 2.1. Experimental Set-Up

**Figure 2:**
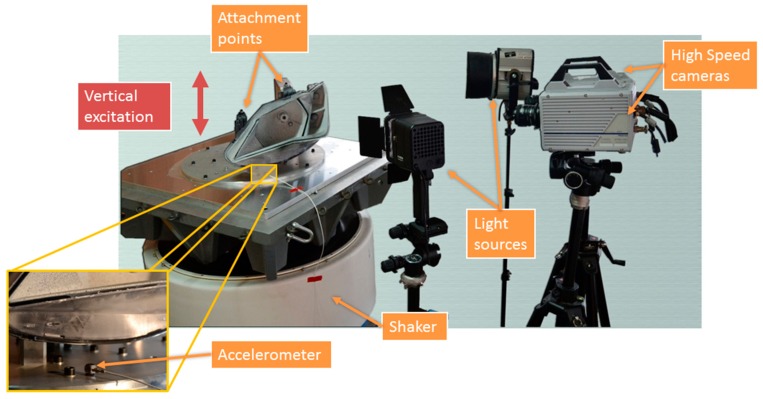
Experimental setup. General arrangement and local zoom on the accelerometer to register excitation.

The setup for experimental modal analysis is presented in Figure 2. The specimen underwent random excitation using an electrodynamic shaker model V2664/DSA4-8k (Data Physics Corporation, San Jose, CA, USA). The element was fixed to a rigid caliber with real mounting conditions. The caliber was attached to the shaker’s armature to transmit the vibration to the attaching points, simulating vertical random excitation in the vehicle. According to the vehicle transit forces, excitation covered a spectrum up to 100 Hz where the most relevant modes occur.

**Figure 3:**
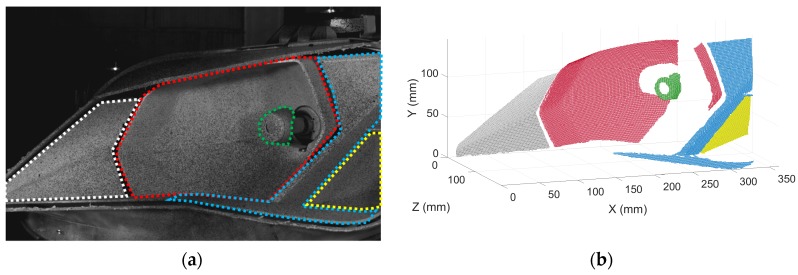
(**a**) Headlamp view from the right-hand camera; (**b**) three-dimensional digitalization of the headlamp using HS 3D-DIC in an unforced state. Areas of interest are shown in color: left area of the bezel (white), main reflector (red), bulbshield (green), right area of the bezel (blue), and TI reflector (yellow).

Two Photron FastCam SA4 high speed cameras (Photron, Tokyo, Japan) were employed, provided with 50 mm f/1.4D focal length Nikon lenses (Nikon Corporation, Tokyo, Japan). The cameras position was arranged to record most of the elements and surfaces. Two light sources were employed to generate uniform light intensity. This was especially important considering the cavities of the headlamp. Figure 3a shows the point of view of the right-hand camera. Hence, it was possible to monitor the left and right areas of the bezel, the main reflector, the bulbshield, and part of the TI reflector. The areas of interest are highlighted in different colors. In these five parts, the full-field behavior was registered by 3D-DIC. To fulfil Nyquist criterion, 250 fps were recorded. Although full image resolution (1024 × 1024 pixels) was available, images were cropped to fit the headlamp shape (1024 × 640 pixels). Hence, the size of the images sets was reduced. The exposure time was 1 ms, a tenth of the maximum frequency period. The excitation was monitored using an accelerometer on the support mount, as shown in Figure 2, representing the shaker’s armature motion. The required synchronization between excitation and response measurements was achieved by a data acquisition system NI USB-6251 DAQ (National Instrument Corporation, Austin, TX, USA). This device digitalized the accelerometer signal according to the cameras’ recording settings.

### 2.2. Transmissibility Functions Using HS 3D-DIC

From the recorded images, the response of the different elements of the system was extracted using HS 3D-DIC [25]. The algorithm divides the region of interest of the images in square facets and performs the tracking of them all. Thus, measurements are provided in a full-field manner. According to the calibration parameters of the stereoscopic cameras system, 3D displacements can be calculated from the tracking of the surface pattern with subpixel precision. A random speckle pattern is required in the specimen surface so that every facet is unique. In Figure 3a, speckle can be appreciated in the evaluated components. It was made by spraying black paint dots over white background with a speckle size between 3–10 pixels [26]. The commercial software Vic-3D (Correlated Solutions Inc., Irmo, South CA, USA) was employed in this study [27]. Facets of 13 pixels with 3 pixels of overlap were employed to increase the spatial resolution. As a result, the 3D digitalization of an unforced state can be seen in Figure 3b. Due to the multiple orientations of the surfaces, none of them was considered as a reference for the coordinate system and an intermediate orientation was employed.

The excitation acceleration and the 3D displacements response were evaluated in the frequency domain to estimate the transfer function in form of transmissibility functions. The calculation of power spectral density of the signals was performed using a frequency lines definition of 1 Hz and anti-leakage Hanning window. In addition, several windows averages were performed to reduce the noise influence.

### 2.3. Procedure for Modal Identification in Base Motion Tests Using the Circle-Fit Approach

The circle-fit approach evaluates the modal parameters on an individual resonance by fitting the peak considering that the shape of the FRF peak in the imaginary plane is circular, as exposed in Appendix A. The adopted methodology to adapt the transmissibility functions to the FRF form for modal identification is based on single degree of freedom assumptions [23]. For a SDOF vibrating system [1], it is possible to obtain a relation between the transmissibility function, T, and the FRF, H

(1) $$H\left\{i\omega \right\}=\frac{T\left\{i\omega \right\}}{{\left(\omega /\omega_{n}\right)}^{2}}\;$$

where ω is the vector of frequencies from the analyzed spectrum and $\omega_{n}$ is the natural frequency of the SDOF system. The complex constant i indicates that H and T are complex functions. This relation is valid for the particular form of transmissibility function that fulfils the following conditions: it has to be non-dimensional, i.e., excitation and response motions are defined by the same magnitude; the response motion is relative to the base (excitation) motion.

According to the experimental procedure, previous modifications must be applied to transmissibility functions to employ Equation (1). The excitation signal in terms of acceleration must be integrated twice to obtain non-dimensional transmissibility functions, i.e., displacements divided by displacements. Finally, HS 3D-DIC measures absolute displacements and thus the excitation must be subtracted to the response to obtain relative displacements. Both operations can be performed in the frequency domain as [23]

(2) $$T\left\{i\omega \right\}=T_{\exp}\left\{i\omega \right\}\cdot {\left(i\omega \right)}^{2}-1$$

where T_exp_ represents the transmissibility functions obtained experimentally with the adopted setup. 

As can be deduced from Equation (1), the natural frequency, $\omega_{n}$, is an input parameter in the conversion procedure. However, it is initially unknown since it is also an output parameter of the circle-fit approach. To work this out, an initial value is proposed, $\omega_{n,0}$, for the conversion defined in Equation (1) to obtain an initial FRF, H_0_. As a result of performing circle fitting in H_0_, the actual natural frequency is obtained since $\omega_{n,0}$, scales the function but does not modify the shape. Thus, no additional iteration is required and the actual FRF, H, can be obtained as

(3) $$H\left\{i\omega \right\}=H_{0}\left\{i\omega \right\}\cdot {\left(\frac{\omega_{n}}{\omega_{n,0}}\right)}^{2}$$

Note that the proposed frequency could be 1 to simplify the calculation.

The circle-fit approach was performed for every DIC measurement point and for every detected resonance. As a result, the information of each resonance involves three full-field matrices of natural frequencies, modal constants and structural damping loss factors. The elements of the matrices correspond to the measurement points of HS 3D-DIC. Hence, the mode shapes were obtained by mapping the modal constant matrices. The natural frequencies and damping ratios were obtained as the mean value. The modal constant was employed as a weight factor to reduce the influence of the noisiest results in the mean value. In particular, damping ratios are provided for each element of the headlamp individually, calculating the mean value in the element’s area. This procedure can be performed in MATLAB using simple routines and an open source circle-fit code [24].

## 3. Results

**Figure 4:**
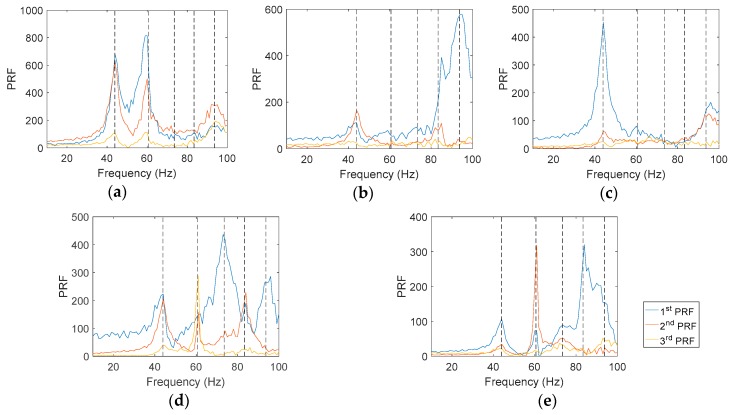
The three first PRFs calculated from transmissibility functions in terms of relative displacements for the different components analyzed in this study: (**a**) right area of the bezel; (**b**) left area of the bezel; (**c**) turn indicator reflector; (**d**) main reflector; and (**e**) bulbshield. Natural frequencies are indicated as vertical dashed lines.

Prior to modal characterization, the number of resonances must be identified through inspection of the transfer functions. Considering the amount of available data and the multiplicity of components, an indicator must be employed to highlight the presence of resonant modes in the analyzed spectrum. In this work, principal response functions (PRFs) were considered for that purpose. PRFs are the result of multiplying the singular values matrix and the left-hand matrix from the single value decomposition of the matrix consisted of the FRFs vectors [1]. The resulting PRFs are a combination of the original FRFs so that the first PRFs contain the most significant information of the modal behavior. For this case, PRFs were obtained for each component of the headlamp using the matrix of transmissibility functions after being adapted according to Equation (2). In Figure 4 the first three PRFs corresponding to the studied elements are shown. Different resonances can be recognized as local maximum values (peaks). Each element shows a different number of peaks with different amplitudes. As a whole, five modes were encountered. The participation of the components in the modes is indicated by the presence or absence of the peaks.

**Figure 5:**
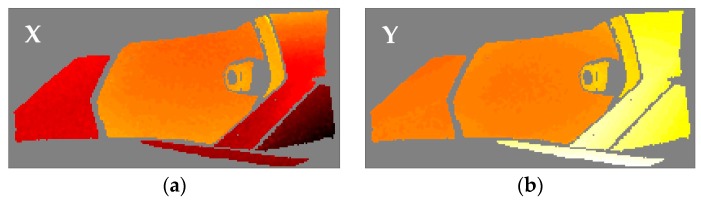

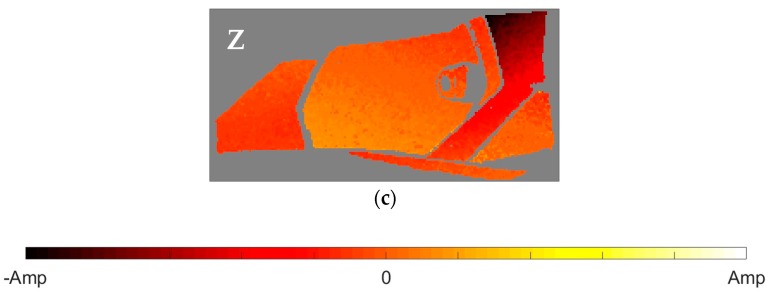
Normalized first mode shape. (**a**) X displacements with amplitude 0.2023; (**b**) Y displacements with amplitude 1; and (**c**) Z displacements with amplitude 0.8014.

**Figure 6:**
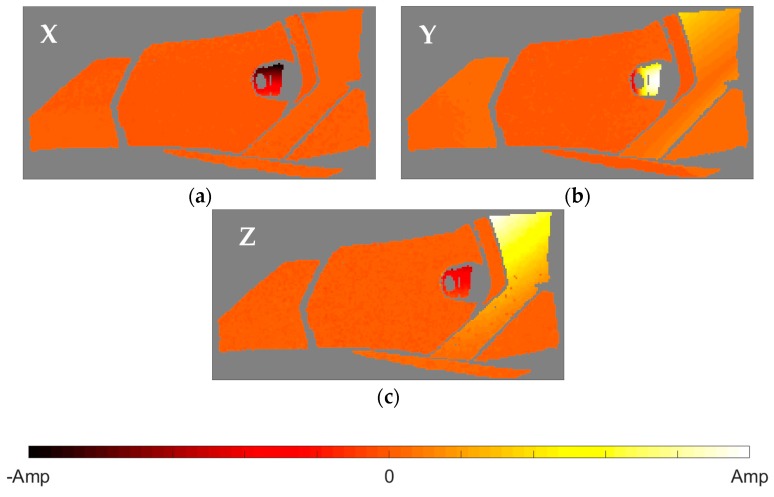
Normalized second mode shape. (**a**) X displacements with amplitude 0.4948; (**b**) Y displacements with amplitude 0.4479; and (**c**) Z displacements with amplitude 1.

**Figure 7:**
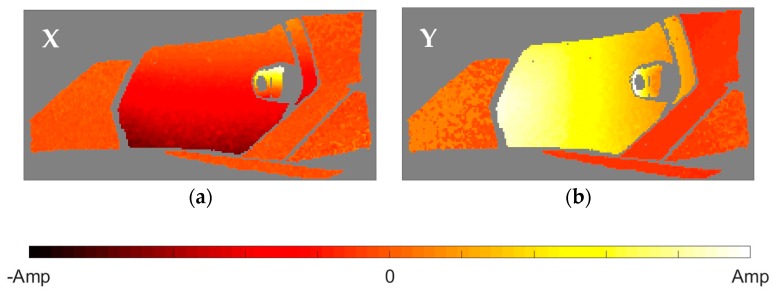
Normalized third mode shape. (**a**) X displacements with amplitude 0.8066; and (**b**) Y displacements with amplitude 1.

**Figure 8:**
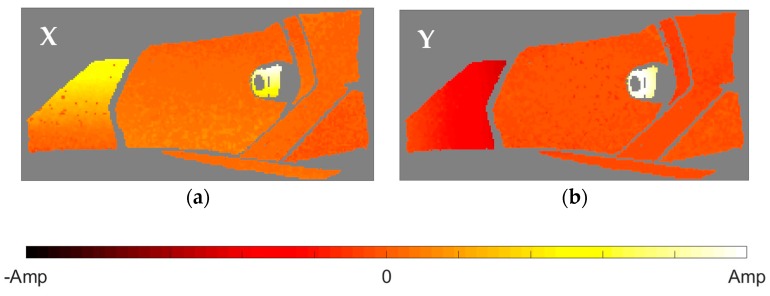
Normalized fourth mode shape. (**a**) X displacements with amplitude 0.5401; and (**b**) Y displacements with amplitude 1.

**Figure 9:**
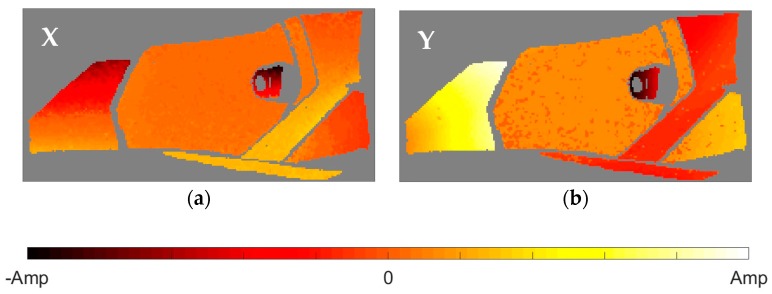
Normalized fifth mode shape. (**a**) X displacements with amplitude 0.6316; and (**b**) Y displacements with amplitude 1.

After the identification of the resonances, the vicinity of the peak is converted into the equivalent FRF form and analyzed through the circle-fit approach. In Table 2 it is shown the natural frequencies and the structural damping ratios for every mode and element of the headlamp. Blank values in damping indicate the absence of a given resonance in the analyzed component, in agreement with PRFs in Figure 4. As can be observed, the damping ratios vary from one component to another for the same mode, rejecting a global approach for energy dissipation. The modal shapes of each resonance in the predominant spatial directions are shown in Figure 5, Figure 6, Figure 7, Figure 8 and Figure 9. The amplitudes were normalized with respect to the maximum value considering all the spatial directions. The normalized amplitudes of each direction are indicated in the figure captions.

The first and the fifth modes involve a global motion of the components as deduced from Table 2 and the mode shapes in Figure 5 and Figure 9, respectively. First mode mainly produces vertical displacements, Y, which are reflected to the other directions. The fifth mode is a more complex combination of motions. The rest of the modes represent local behavior of the components. The second mode (Figure 6) consists in a flapping of the upper region of the right area of the bezel, highlighted in Z displacements, and a torsion of the bulbshield as can be deduced from the X and Y directions. In comparison with these two elements, the motion of the reflector is negligible but resonance is present as seen in Figure 4d. The third mode (Figure 7) is a laterally nodding of the reflector and the bulbshield as a whole. In the fourth mode (Figure 8) the bulbshield is nodding and the left area of the bezel is swaying. In the last three modes, the out-of-plane displacements, Z, were negligible and they are not exposed here.

Furthermore, the full-field multi-component analysis reveals particularities in the damping ratios. The bulbshield damping shows some particular aspects since it is the only metallic component (Table 1). To evaluate the damping ratios with respect to the rest of the polymeric elements, it is also necessary to take into account the mode shape. For instance, in the third mode, the motion of the bulbshield occurs mutually with the main reflector, as previously indicated. Thus, damping values of both elements, 8.18% for the bulbshield and 7.97% for the main reflector, are similar. Nevertheless, the torsional motion of the bulbshield in the second mode is independent from the reflector motion since, although it is in resonance, reflector amplitude is much lower regarding the two other elements involved. Consequently, the bulbshield behaves as a separated element due to the uncoupling from the reflector motion and the damping is proper of a metallic element, 1.14%. As usual, it is a lower value compared with plastic components (~6% for the mentioned mode), although it could vary depending on the stress amplitude [28,29]. The same reason can be adopted to explain the observed behavior for the fourth mode where the bulbshield nodding, with 3.42% of structural damping, is also uncoupled from the reflector, at 6.88%.

## 4. Conclusions

HS 3D-DIC has been employed to perform a full-field experimental modal analysis in automotive multi-component system where different materials are present. Modal identification was conducted using the circle-fit approach in base motion tests with a methodology that takes into account the relation between transmissibility functions and frequency response functions. 

The potential of the methodology for these systems was demonstrated. The complex shape, and also the light weight of the system, make it difficult to employ traditional sensors or alternatives techniques like SLDV. In fact, with such a remarked 3D behavior observed in this specimen, it would be required an expensive 3D SLDV system. With this methodology, it was possible to evaluate the modal behavior of each element, detecting which elements participate in each mode, the deformations that result, and interactions between them. The methodology also made it possible to individually characterize the energy dissipation through the structural damping ratio. In this sense, the materials features could be found out according to the typology of the materials. 

Although the actual modal behavior may slightly differ due to the absence of the external lens, with this methodology it is achieved a significant advance towards a deeper experimental knowledge of headlamps behaviors that can be extend to other multi-material multi-component systems, supplying numerical models with invaluable information for validation.

## Figures and Tables

**Table 1:** Materials of the elements of the system analyzed in this study.

Element	Material
Bezel	Polycarbonate
Main reflector	Thermoset BMC
TI reflector	Polycarbonate
Bulbshield	Sheet metal steel

**Table 2:** Natural frequencies and structural damping ratio of the five modes of the multi-component system.

Mode	Natural Frequency (Hz)	Structural Damping (%)				
		Bezel’s Right Area	Bezel’s left Area	TI Reflector	Main Reflector	Bulbshield
1	43.92	8.17	6.68	8.38	7.68	7.87
2	60.56	6.24	-	-	6.61	1.14
3	73.45	-	-	-	7.97	8.18
4	83.42	-	3.72	-	6.88	3.42
5	93.68	5.98	6.24	4.99	4.76	7.95

**Figure A1:**
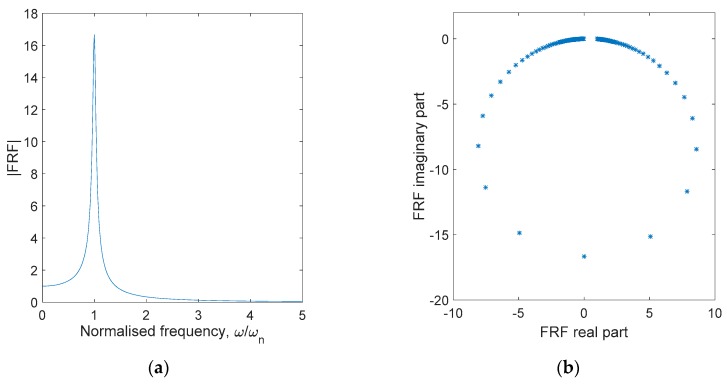
Non-dimensional FRF of a SDOF system with a structural damping of 0.06 represented (**a**) in magnitude and (**b**) in the complex plane.

**Figure A2:**
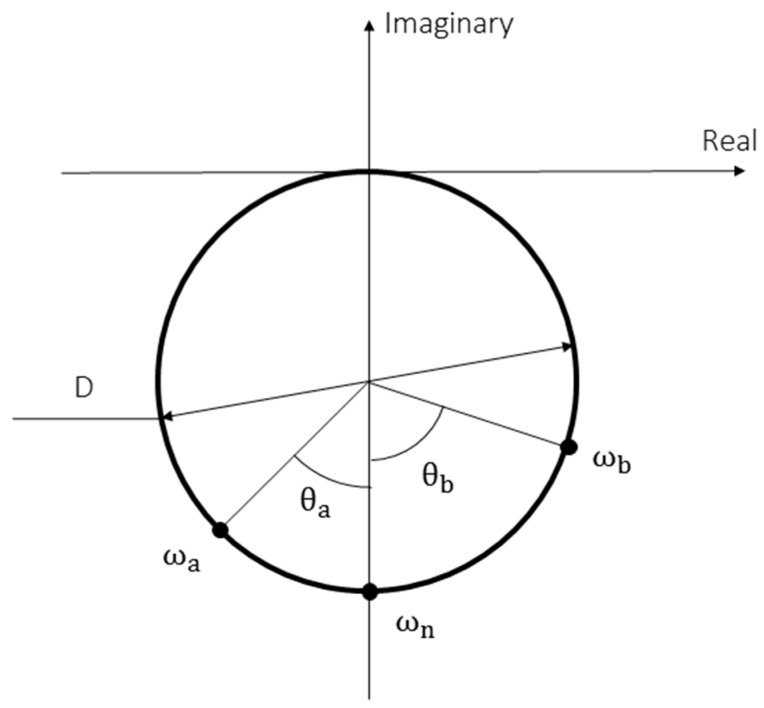
FRF plotted in the complex plane to show the relation between modal and geometrical parameters.

## Appendix

In this appendix, the fundamentals of the circle-fit method are briefly described. The full method employed in this work can be found in the literature [1]. In an SDOF system with structural damping, the FRF in its non-dimensional form is defined by Equation (A1). In Figure A1, the FRF corresponding to a system with a structural damping of 0.06 is plotted in magnitude (a) and in the complex plane (b). It can be observed that this function describes a perfect circumference.

(A1) $$H\left\{\omega \right\}=\frac{1}{1-{\left(\frac{\omega }{\omega_{n}^{2}}\right)}^{2}+i\eta }\;,$$

Theoretically, a relation exists between the modal and the circumference geometrical parameters shown in Figure A2. The natural frequency, $\omega_{n}$, is determined as that with maximum response, which implies the furthest point from the origin. The structural damping, η, can be obtained from the frequency, ω, and the geometrical position defined by the angle, θ, of any point above, a, and any below the resonance, b. Both parameters together with the modal constant, A, (employed to define the mode shape) are related with the circumference diameter, D, as observed in Equation (A3).

Experimentally, a set of data points are selected at the vicinity of the peak to fit them to a circle using the least-squares method. The natural frequency is obtained from the resulting curve. As the actual curve is not expected to be a perfect circumference, the damping ratio is calculated as the mean value of the damping ratios obtained from the combination of every point above and below the resonance in the selected region using Equation (A2). Finally, the modal constant is obtained from Equation (A3).

(A2) $$\eta =\frac{\omega_{a}^{2}-\omega_{b}^{2}}{\omega_{n}^{2}\left(\tan\frac{\theta_{a}}{2}+\tan\frac{\theta_{b}}{2}\right)}$$

(A3) $$D=\frac{\left|A\right|}{\omega_{n}^{2}\;\eta }$$
